# County-level variation in healthcare coverage and ischemic heart disease mortality

**DOI:** 10.1371/journal.pone.0292167

**Published:** 2024-01-26

**Authors:** Ramzi Ibrahim, Adam Habib, Kristina Terrani, Soumiya Ravi, Chelsea Takamatsu, Mohammed Salih, João Paulo Ferreira

**Affiliations:** 1 Department of Medicine, University of Arizona Tucson, Tucson, Arizona, United States of America; 2 University of Arizona College of Medicine–Tucson, Tucson, Arizona, United States of America; 3 The Heart Hospital, Baylor University Medical Center, Plano, Texas, United States of America; Icahn School of Medicine at Mount Sinai, UNITED STATES

## Abstract

**Background:**

Healthcare coverage has been shown to have implications in the prevalence of coronary artery disease. We explore the impact of lack of healthcare coverage on ischemic heart disease (IHD) mortality in the US.

**Methods:**

We obtained county-level IHD mortality and healthcare coverage data from the CDC databases for a total of 3,119 US counties. The age-adjusted prevalence of current lack of health insurance among individuals aged 18 to 64 years were obtained for the years 2018 and 2019 and were placed into four quartiles. First (Q1) and fourth quartile (Q4) had the least and highest age-adjusted prevalence of adults without health insurance, respectively. IHD mortality rates, adjusted for age through the direct method, were obtained for the same years and compared among quartiles. Ordinary least squares (OLS) regression for each demographic variable was conducted with the quartiles as an ordinal predictor variable and the age-adjusted mortality rate as the outcome variable.

**Results:**

We identified a total of 172,942 deaths related to ischemic heart disease between 2018 and 2019. Overall AAMR was higher in Q4 (92.79 [95% CI, 92.35–93.23]) compared to Q1 (83.14 [95% CI, 82.74–83.54]), accounting for 9.65 excess deaths per 100,000 person-years (*slope* = 3.47, p = 0.09). Mortality rates in Q4 for males (126.20 [95% CI, 125.42–126.98] and females (65.57 [95% CI, 65.08–66.05]) were higher compared to Q1 (115.72 [95% CI, 114.99–116.44] and 57.48 [95% CI, 57.04–57.91], respectively), accounting for 10.48 and 8.09 excess deaths per 100,000 person-years for males and females, respectively. Similar trends were seen among Hispanic and non-Hispanic populations. Northeastern, Southern, and Western regions had higher AAMR within Q4 compared to Q1, with higher prevalence of current lack of health insurance accounting for 49.2, 8.15, and 29.04 excess deaths per 100,000 person-years, respectively.

**Conclusion:**

A higher prevalence of adults without healthcare coverage may be associated with increased IHD mortality rates. Our results serve as a hypothesis-generating platform for future research in this area.

## Introduction

Cardiovascular disease remains a leading cause of morbidity and mortality in the United States (US). Many risk factors exist, including socioeconomic status, that have significant implications in the prevalence, treatment, and prognosis of cardiovascular disease. Among the socioeconomic factors implicated in population health, health insurance is an underpinning to accessibility to healthcare. Lack of healthcare coverage has increased in recent years and has impacted all-cause mortality, cardiovascular death, and cancer mortality [1].

Absence of healthcare coverage has been linked to a higher prevalence of coronary artery disease and challenges in its management [2, 3]. Yet, the correlation between county-specific health insurance rates and mortality due to ischemic heart disease (IHD) remains under-explored. Health insurance is a pivotal factor in ensuring healthcare accessibility, particularly in managing chronic diseases like IHD, which necessitate regular health consultations and continuous care. In the context of an acute coronary syndrome, as illustrated during the COVID-19 pandemic, immediate access to healthcare is a critical determinant of mortality and morbidity associated with ischemic heart diseases [4]. For instance, delayed access to care or late presentation often leads to worse health outcomes. Factors contributing to such delays often include fear of financial burdens and barriers to care.

Therefore, our study focuses primarily on IHD. We aim to delve deeper into whether a deficiency in healthcare coverage influences mortality from IHD across all US counties. Our exploration encompasses an overall view as well as stratified analysis amongst different sex, racial, and geographical subgroups.

## Methods

We obtained mortality data from the Centers for Disease Control and Prevention (CDC) Wide-ranging ONline Data for Epidemiologic Research (WONDER) database from the years 2018 and 2019 [5]. Data was obtained through the National Vital Statistics System, which captures all mortality data via death certificate information. Death certificate information includes underlying causes of death, multiple causes of death, and demographic information. The World Health Organization defined the underlying cause of death as the disease process that initiated the events that led to death or if it led directly to death. The CDC defined the multiple causes of death as the disease processes that contributed to mortality and can be up to twenty different causes. All diagnoses are based on the *International Classification of Diseases*, *Tenth Revision* (ICD10). Demographic information within the death certificates included sex (i.e. male and female), race (i.e. Hispanic versus non-Hispanic, White, Black, American Indian/Alaska Native, and Asian/Pacific Islander), and geographic (i.e. U.S. Census Regions—Northeast, Midwest, South, And West) information. Additionally, US counties were aggregated by metropolitan and non-metropolitan subtypes with use of the National Center for Health Statistics 2013 Urban–Rural Classification Scheme.

The CDC PLACES database provides community-level information regarding chronic disease measures and factors implemented with social determinants of health [6]. This database is an expansion of the original 500 Cities Project which initially began in 2015. To date, it provides county-, census tract-, and place-level data using small area estimation models to provide data regarding 29 distinct chronic disease related measures in the US (**Table 1**). Specifically, we obtained healthcare coverage information utilizing the 2021 release of county-level data which is based on the Behavioral Risk Factor Surveillance System information from 2018 and 2019.

**Table 1:** Table of the CDC PLACES county-level data including four underlying themes encompassing 29 distinct chronic disease/social determinants of health-related measures.

Healthcare Outcomes	Prevention	Health Risk Behaviors	Health Status
Stroke among adults ≥18 years	Older adults aged ≥65 years who are up to date on a core set of clinical preventive services by age and sex	Sleeping less than 7 hours among adults aged ≥18 years	Fair or poor self-rated health status among adults aged ≥18 years
All teeth lost among adults aged ≥65 years	Fecal occult blood test, sigmoidoscopy, or colonoscopy among adults aged 50–75 years	No leisure-time physical activity among adults aged ≥18 years	Physical health not good for ≥14 days among adults aged ≥18 years
Obesity among adults aged ≥18 years	Cervical cancer screening among adult women aged 21–65 years	Current smoking among adults aged ≥18 years	Mental health not good for ≥14 days among adults aged ≥18 years
Diagnosed diabetes among adults aged ≥18 years	Mammography use among women aged 50-74 years	Binge drinking among adults aged ≥18 years	
Depression among adults aged ≥18 years	Cholesterol screening among adults aged ≥18 years		
Coronary heart disease among adults ≥18 years	Taking medicine for high blood pressure control among adults aged ≥18 years with high blood pressure		
Chronic obstructive pulmonary disease among adults aged ≥18 years	Visits to dentist or dental clinic among adults aged ≥18 years		
Chronic kidney disease among adults aged ≥18 years	Visits to doctor for routine checkup within the past year among adults aged ≥18 years		
High cholesterol among adults aged ≥18 years who have been screened in the past 5 years	Current lack of health insurance among adults aged 18–64 years		
Cancer among adults aged ≥18 years			
High blood pressure among adults aged ≥18 years			
Current asthma prevalence among adults aged ≥18 years			
Arthritis among adults aged ≥18 years			

### Statistical analysis

A model-based estimate for age-adjusted prevalence of current lack of health insurance among adults aged 18 to 64 years for all 3,119 US counties was queried from the CDC PLACES database. Four quartiles were used for rankings of age-adjusted prevalence of current lack of health insurance. First quartile included 780 counties with an age-adjusted prevalence between 7.9–13.4 [Q1], followed by 780 counties with an age-adjusted prevalence of 13.4–16.6 [Q2], 780 counties with an age-adjusted prevalence of 16.6–21.2 [Q3], and finally 779 counties with an age-adjusted prevalence of 21.2–56.6 [Q4]. Counties within Q1 had the least age-adjusted prevalence of adults without health insurance, and Q4 included the highest of age-adjusted prevalence of adults without insurance.

In the US, every residential address is given a 15-digit geographic identifier (GEOID) comprising a 3-digit county code, 5-digit census tract code, and/or 4-digit census block code. This GEOID enabled us to link each county, among the quartiles based on healthcare prevalence, to the mortality data within that specific county. With this information, we obtained mortality data related to ischemic heart diseases (ICD10: I20-I25) as the underlying cause of death from the years 2018 and 2019 for each quartile, cumulatively and stratified by sex, racial, and geographic subgroups. Counties were included in our analysis if death counts were above 20 during our pre-specified years. Counties were excluded if their death rates represented zero to twenty persons given the confidentiality constraints from the National Center for Health Statistics.

Age-adjusted mortality rates per 100,000 population were calculated with corresponding 95% confidence intervals (CI). The age-adjusted mortality rates were adjusted to the year 2000 US population using the direct method. Adjustment for other co-variates were not completed due to lack of other individual-level patient data availability. We also calculated rate differences, depicted as an excess or fewer deaths per 100,000 person-years, by comparing Q4 and Q1. We conducted an ordinary least squares (OLS) regression analysis for each demographic variable as regression diagnostics did not reveal major violations of OLS assumptions. We used the quartiles as the ordinal predictor variable by assigning them numerical values and used the age-adjusted mortality as our outcome variable. Slopes represent changes in age-adjusted mortality rates for each step up in quartile with a positive slope indicating increasing mortality and decreasing slope indicating decreasing mortality. A p-value less than 0.05 was considered statistically significant. Patient-level data were not available in the repositories; therefore, we did not account for potential confounders.

Statistical software (Stata Statistical Software: Release 17.0; StataCorp LLC) was used for data input and analysis.

## Results

**Fig 1:**
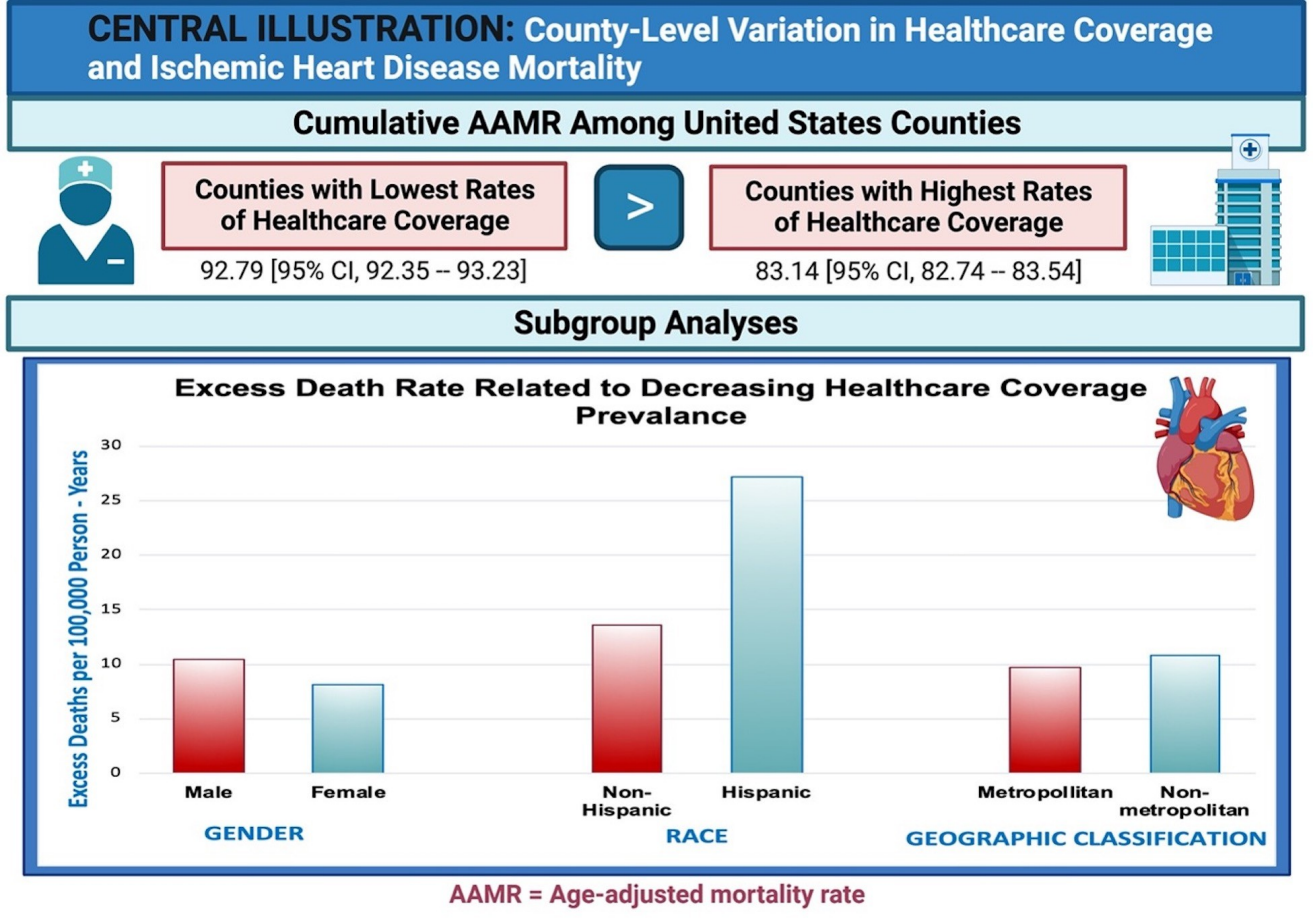
Central illustration. Attached separately.

We identified a total of 172,942 deaths related to ischemic heart disease between 2018 and 2019 (**Fig 1**). Absolute death counts, population size, and crude and age-adjusted mortality rates with respective 95% confidence intervals are depicted in **S1 Table**. Side by side comparisons of age-adjusted mortality rates by insurance quartiles are depicted in **Table 2** and results of regression analyses in **Table 3**. Cumulative age-adjusted mortality rates were significantly higher within Q4 (92.79 [95% CI, 92.35–93.23]) as compared to Q1 (83.14 [95% CI, 82.74–83.54]), with higher prevalence of current lack of health insurance accounting for 9.65 excess deaths per 100,000 person-years (*slope* = 3.47, p = 0.09). Sex analyses revealed higher age-adjusted mortality rates among males and females in Q4 (126.20 [95% CI, 125.42–126.98] and 65.57 [95% CI, 65.08–66.05], respectively) compared to mortality rates within Q1 (115.72 [95% CI, 114.99–116.44] and 57.48 [95% CI, 57.04–57.91], respectively). Higher prevalence of current lack of health insurance accounted for 10.48 and 8.09 excess deaths per 100,000 person-years for males (*slope* = 3.79, p = 0.10) and females (*slope* = 2.92, p = 0.09), respectively.

**Table 2:** Age-adjusted mortality rates for ischemic heart diseases across US counties aggregated by healthcare coverage quartiles, 2018–2019. Table depicting the AAMR, rate excess, and respective 95% confidence intervals for all insurance quartiles.

Population	Q1 (95% CI)	Q2 (95% CI)	Q3 (95% CI)	Q4 (95% CI)	Rate Excess
Total–AAMR.	83.14 (82.74–83.54)	88.11 (87.67–88.54)	93.84 (93.42–94.27)	92.79 (92.35–93.23)	9.65 excess deaths
Male–AAMR.	115.72 (114.99–116.44)	121.48 (120.70–122.25)	127.91 (127.15–128.67)	126.20 (125.42–126.98)	10.48 excess deaths
Female–AAMR.	57.48 (57.04–57.91)	61.65 (61.17–62.12)	66.57 (66.10–67.04)	65.57 (65.08–66.05)	8.09 excess deaths
Hispanic–AAMR.	47.35 (45.48–49.22)	58.55 (57.05–60.05)	70.70 (69.47–71.93)	74.48 (73.56–75.40)	27.13 excess deaths
Non-Hispanic–AAMR.	83.99 (83.58–84.40)	89.79 (89.34–90.24)	96.25 (95.80–96.71)	97.63 (97.13–98.13)	13.64 excess deaths
Black–AAMR.	96.53 (94.60–98.45)	105.97 (104.32–107.61)	106.84 (105.57–108.11)	96.93 (95.69–98.17)	0.4 excess deaths
White–AAMR.	84.73 (84.31–85.16)	88.54 (88.07–89.00)	93.68 (93.21–94.15)	93.82 (93.33–94.30)	9.09 excess deaths
Asian/Pacific Islander–AAMR.	44.78 (43.55–46.00)	49.08 (47.65–50.50)	64.18 (62.64–65.73)	50.26 (48.51–52.01)	5.48 excess deaths
American Indian/Alaska Native–AAMR.	62.93 (57.71–68.14)	65.05 (60.79–69.31)	68.11 (64.72–71.50)	53.18 (49.69–56.67)	9.75 fewer deaths
Metropolitan–AAMR.	79.89 (79.46–80.32)	84.79 (84.32–85.26)	91.08 (90.62–91.53)	89.60 (89.14–90.07)	9.71 excess deaths
Non-metropolitan–AAMR.	99.72 (98.65–100.80)	103.23 (102.14–104.33)	108.78 (107.64–109.92)	110.52 (109.30–111.74)	10.80 excess deaths
Northeast–AAMR.	87.92 (87.23–88.60)	92.15 (91.15–93.15)	128.75 (127.04–130.46)	137.12 (132.91–141.33)	49.20 excess deaths
Midwest–AAMR.	86.09 (85.38–86.80)	100.36 (99.58–101.15)	93.36 (92.24–94.48)	82.88 (81.37–84.40)	3.21 fewer deaths
South–AAMR.	84.66 (83.67–85.65)	89.06 (88.03–90.10)	91.71 (91.08–92.34)	92.81 (92.31–93.31)	8.15 excess deaths
West–AAMR.	64.21 (63.33–65.09)	69.47 (68.75–70.19)	87.72 (87.01–88.44)	93.25 (92.12–94.39)	29.04 excess deaths

• Rate excess or fewer deaths are reported as comparisons between Q4 and Q1. Values are per 100,000 person-years.

• Abbreviations: AAMR, age-adjusted mortality rates; CI, confidence interval; US, United States; Q, quartile.

**Table 3:** Ordinary least regression analysis results. Quartiles designated as the ordinal predictor variable and age-adjusted mortality as outcome variable. Table includes regression analysis results of identifying the association between age-adjusted mortality among increasing social vulnerability quartiles.

Variable	Slope	Intercept	R-squared	p-value
All	3.47	80.80	0.83	0.09
Female	2.92	55.52	0.83	0.09
Male	3.79	113.36	0.80	0.10
American Indian/Alaska Native	-2.62	68.87	0.27	0.48
Asian/Pacific Islander	3.15	44.19	0.23	0.52
Black/African American	0.21	101.05	0.002	0.95
White	3.24	82.09	0.91	0.05
Hispanic	9.35	39.39	0.96	0.02
Non-Hispanic	4.74	80.07	0.95	0.03
Northeast	18.42	65.44	0.90	0.05
Midwest	-1.66	94.83	0.08	0.72
South	2.71	82.78	0.93	0.04
West	10.54	52.32	0.94	0.03
Metropolitan	3.54	77.49	0.81	0.10
Non-metropolitan	3.80	96.08	0.97	0.02

Non-Hispanic populations encountered higher age-adjusted mortality rates within Q4 compared to Q1, with higher prevalence of current lack of health insurance accounting for 13.64 excess deaths per 100,000 person-years (*slope* = 4.74, p = 0.03). Hispanic populations also encountered higher age-adjusted mortality rates within Q4 compared to Q1, with higher prevalence of current lack of health insurance accounting for 27.13 excess deaths per 100,000 person-years (*slope* = 9.35, p = 0.02). White populations had an age-adjusted mortality rate of 93.82 [95% CI, 93.33–94.30] in Q4 compared to 84.73 [95% CI, 84.31–85.16] in Q1, with higher prevalence of current lack of health insurance accounting for 9.09 excess deaths per 100,000 person-years (*slope* = 3.24, p = 0.05). Asian populations had a higher age-adjusted mortality rate in Q4 (50.26 [95% CI, 48.51–52.01]) compared to Q1 (44.78 [95 CI, 43.55–46.00]), with higher prevalence of current lack of health insurance accounting for 5.48 excess deaths per 100,000 person-years (*slope* = 3.15, p = 0.52). American Indian/Alaska Native (*slope* = -2.62, p = 0.48) and Black populations (*slope* = 0.21, p = 0.95) did not have higher mortality rates in US counties with a larger uninsured population.

Geographic analyses revealed higher age-adjusted mortality rates among majority of US census regions that include a greater prevalence of current lack of health insurance. For example, the Northeast had an age-adjusted mortality rate of 137.12 [95% CI, 132.91–141.33] in Q4 compared to 87.92 [95% CI, 87.23–88.60] in Q1, with higher prevalence of current lack of health insurance accounting for 49.2 excess deaths per 100,000 person-years (*slope* = 18.42, p = 0.05). The South and West also had higher age-adjusted mortality rates within Q4 (92.81 [95% CI, 92.31–93.31]) and 93.25 [95% CI, 92.12–94.39], respectively) compared to counties in Q1 (84.66 [95% CI, 83.67–85.65] and 64.21 [95% CI, 63.33–65.09], respectively), with higher prevalence of current lack of health insurance accounting for 8.15 (*slope* = 2.71, p = 0.04) and 29.04 (*slope* = 10.54, p = 0.03) excess deaths per 100,000 person-years, respectively. There was no increased mortality within Q4 compared to Q1 for US counties in the Midwest (*slope* = -1.66, p = 0.72). Age-adjusted mortality rates in metropolitan regions in US counties among Q4 regions was 89.60 [95% CI, 89.14–90.07]), compared to Q1 which was 79.89 [95% CI, 79.46–80.32]), with higher prevalence of current lack of health insurance accounting for 9.71 excess deaths per 100,000 person-years (*slope* = 3.54, p = 0.10). Age-adjusted mortality rates was 110.52 [95% CI, 109.30–111.74] in Q4 for non-metropolitan regions as compared to 99.72 [95% CI, 98.65–100.80] in Q1, with higher prevalence of current lack of health insurance accounting for 10.8 excess deaths per 100,000 person-years (*slope* = 3.80, p = 0.02).

## Discussion

Our analysis identified that majority of US counties with higher rates of uninsured prevalence is had higher rates of IHD mortality rates based on descriptive epidemiology, mainly independent of race, sex, or geographic residence. However, increasing quartiles related to lack of healthcare coverage was not statistically associated with higher IHD mortality. This provides valuable epidemiological insight into overall IHD mortality trends in the US with strong health policy implications.

Our findings present contemporary analyses that represent a nationwide disparity. Socioeconomic disadvantages encompass poor healthcare inaccessibility and financial strains, leading to a greater risk of cardiovascular disease and associated risk factors including hypertension, diabetes mellitus, obesity, and smoking [7]. At the county-level, healthcare outcomes are likely to be impacted by varying rates of healthcare coverage, employment opportunities, racial minority status, language and transportation barriers, and immigration status. In congruence, previous analyses revealed that treatment of cardiovascular disease risk factors are lower in uninsured populations and are more likely to have coronary artery disease [2]. These healthcare coverage gaps are more likely to lead to worse short-term outcomes, poor quality of care, and are less likely to receive evidence-based therapies and invasive cardiac procedures [2, 3, 8].

Racial analyses revealed that both Hispanic and non-Hispanic populations were affected by higher ischemic heart disease mortality in regions with decreasing healthcare coverage. Hispanic populations had a 27.13 excess death rate per 100,000 person-years, representing one of the highest impacted populations in our analyses. Hispanic populations are more likely to lack healthcare coverage and are more likely to lose their coverage compared to non-Hispanic populations, likely a result of language and educational status barriers [9]. Interestingly, we did not observe a statistically significant difference in ischemic heart disease mortality in Black populations, stratified by healthcare coverage prevalence. Contrarily, previous studies have identified that uninsured Black adults have worse outcomes associated with cardiovascular disease [9–11]. A potential explanation to our finding is based on results from a recent cross-sectional analysis in 2017, revealing that Black populations were faster to gain healthcare coverage than other racial groups [9]. In this same study, White populations were expected to live approximately eight years without insurance before the age of 65 years, as opposed to Black individuals who were expected to live twelve years without health coverage, explaining the increased mortality seen among White populations in our study. Lastly, Asian communities in the US were found to have higher rates of loss of healthcare coverage after the age of 40 years and during young adulthood. This loss of coverage may hinder accessibility to care and, therefore, impact comorbidity and mortality [9].

Geographic variability in ischemic heart diseases exists, largely contributed by varying rates of healthcare coverage. For example, US census regions including the West and South showed that populations with a lack of healthcare coverage were negatively impacted by higher ischemic heart disease mortality; however, the Midwest and Northeast were not affected. Multiple geographic characteristics predict the likelihood of lack of healthcare coverage. For example, proportion of racial minority residents in a US county, poverty, unemployment, Republican voting patterns, and non-metropolitan regions predicts higher uninsured population rates [12]. Majority of US counties with the highest lack of healthcare coverage were located along the US-Mexico border, as many of the available job opportunities in industries in this region do not offer employer sponsored insurance coverage, offer too low of wages to afford insurance premiums, and are impacted by both language and immigration status [13–15]. Additionally, US counties overlapping those of tribal nations have previously shown high uninsured rates; however, access to healthcare within these counties are highly accessed through the Indian Health Services. Lastly, state and county-level variation in voting patterns may have an impact on political ideologies and, therefore, impact the county-level policies regarding healthcare coverage [12, 16–18]. For example, democratic political traditions may lead to increasing healthcare coverage and higher investment rates into public services, whereas conservative parties may lead to lower investment rates into public services and higher dependency on privatized healthcare coverage [12].

Healthcare spending has seen a marked rise in recent years, mainly fueled by a surge in ambulatory care costs and retail pharmaceutical spending. The claims for cardiovascular disease screening and monitoring strategies under the Medicare fee-for-service scheme have also significantly risen, further escalating healthcare costs. Hence, public insurance, followed by private insurance and out-of-pocket expenses, accounted for most of the healthcare expenses related to cardiovascular diseases in recent years [19]. Obtaining and keeping healthcare coverage in the US is a dynamic process; frequent losses of coverage, affordability of care, and accessibility to appropriate healthcare coverage plans have negative consequences on population health. Previous studies have revealed the impact of healthcare coverage in significant healthcare delivery hindrances. A recent systematic review and meta-analysis revealed poor health outcomes and higher mortality rates related to malignancies in patients with a lack of healthcare coverage [20]. Types of healthcare coverage were also shown to impact infection rates and mortality outcomes due to COVID-19 based on comparison of Medicaid expansion and non-expansion states [21]. Searching for healthcare providers that fall within a new healthcare coverage plan may be difficult. Additionally, the patient-provider relationship may be lost if healthcare coverage changes, preventing the benefits of continuity of care. Lastly, anticipation of losing healthcare coverage at a future point may impact the quality of care one receives by preferring shorter-term solutions to their problems. For example, this may include fewer indicated surgical procedures, lack of referral to specialists, and treatment plans that require fewer follow up visits. Unstable healthcare plans are a component of multiple economic and social factors, translating into disparities on healthcare delivery [9].

In the previous decade, healthcare expansion has been a major topic of healthcare reform. Policy makers have called and requested legislation to expand coverage multiple times to develop a universal public option for healthcare coverage. This involves increasing and expanding eligibility to subsidies with a hopeful goal of reducing out of pocket costs, expansion Medicare coverage to all US adults, and reducing the Medicare eligibility age. Additionally, these plans would ultimately decrease the rate of enrollment into private plans and employer sponsored insurance options, increase access to having a personal healthcare provider, and increase stability in insurance coverage [22]. In the current setting, healthcare coverage remains widely heterogenous in the US and has major clinical implications.

Our study has limitations. Obtaining mortality data from death certificate information in the US has inherent limitations given the ambiguity of completing death certificates. For example, an individual death related to cardiac arrest secondary to ischemic heart disease may be documented as “cardiac arrest [ICD10: I46]” as the underlying cause of death, rather than ischemic heart disease. Therefore, misclassification or under-reporting of data using death certificate information is possible. Additionally, we clearly depict an association between healthcare coverage rates and ischemic heart disease mortality; however, due to the cross-sectional design of this study, we are unable to establish causality. Nonetheless, our limitations are unlikely to explain the existing disparities identified in our analyses. It should also be noted that our study is limited by its inability to account for household income and other co-variates. This is particularly significant as individuals in lower socioeconomic brackets who lack healthcare coverage may experience more pronounced effects compared to those in higher socioeconomic brackets. Lastly, OLS assumptions were mainly met through diagnostic regressions. However, potential heteroscedasticity and deviations from normality were observed in certain subpopulations. These limitations should be taken into consideration upon interpretation of our results as cross-sectional observational data is hypothesis-generating, requiring future work in this area to validate our findings.

Our study is strengthened by the broad capture of mortality rates across the US. The CDC WONDER database obtains death certificate information from the National Vital Statistics System which registers more than 99% of deaths in the US, allowing our study to evaluate IHD mortality on a larger scale [23]. Although previous analyses have identified certain outcomes related to the lack of healthcare coverage on IHD mortality, our study goes beyond these findings by identifying specific subpopulations that are disproportionately affected. Ultimately, we identified using descriptive epidemiology that higher rates of IHD mortality was higher in regions with greater lack of healthcare coverage. However, given the lack of statistical significance based on the quartile variation in IHD mortality, limits the external validity of our findings. Nonetheless, our framework enables both researchers and healthcare delivery systems to leverage the depression data available through the CDC PLACES database to pinpoint populations that are vulnerable to IHD mortality.

## Conclusion

In conclusion, a lack of healthcare coverage may play a crucial role in mortality rates related to ischemic heart disease and encumbers delivery of quality care for uninsured US residents. Our cross-sectional analyses using national data from publicly available repositories serves as a hypothesis-generating platform for future research in this area. Further data is needed to validate our observations.

## Supporting information

S1 Table Table includes absolute death count, population size, crude mortality rates (95% CI), and age-adjusted mortality rates (95% CI), stratified by healthcare coverage quartiles (Q1 to Q4). (DOCX)

## Abbreviations

AAMR: age-adjusted mortality rate
CDC: Centers for Disease Control and Prevention
CI: confidence interval
CVD: Cardiovascular disease
ICD10: International Classification of Diseases-Tenth Revision
IHD: ischemic heart diseases
US: United States
WONDER: Wide-Ranging Online Data for Epidemiologic Research; Tenth Revision
Q: quartile
