# Supplementary material for: County-level variation in healthcare coverage and ischemic heart disease mortality
Source: PLoS One. 2024 Jan 26;19(1):e0292167. doi: 10.1371/journal.pone.0292167 (PMC10817196; doi:10.1371/journal.pone.0292167)
Supplement: S1 Table — (DOCX) [file pone.0292167.s001.docx]

**Table S1**. Table includes absolute death count, population size, crude mortality rates (95% CI), and age-adjusted mortality rates (95% CI), stratified by healthcare coverage quartiles (Q1 to Q4).

|  | **Variable** | **Deaths** | **Population** | **Crude Rate (95% CI)** | **Age-Adjusted Mortality Rate (95% CI)** |
| --- | --- | --- | --- | --- | --- |
| **Q1** | All | 172,942 | 157,200,243 | 110.01 (109.5-110.53) | 83.14 (82.74-83.54) |
|  | Female | 70,867 | 79,613,814 | 89.01 (88.36-89.67) | 57.48 (57.04-57.91) |
|  | Male | 102,075 | 77,586,429 | 131.56 (130.76-132.37) | 115.72 (114.99-116.44) |
|  | American Indian/Alaska Native | 620 | 1,270,813 | 48.79 (44.95-52.63) | 62.93 (57.71-68.14) |
|  | Asian/Pacific Islander | 5,251 | 12,558,728 | 41.81 (40.68-42.94) | 44.78 (43.55-46) |
|  | Black/African American | 10,275 | 13,591,617 | 75.6 (74.14-77.06) | 96.53 (94.6-98.45) |
|  | White | 156,796 | 129,779,085 | 120.82 (120.22-121.42) | 84.73 (84.31-85.16) |
|  | Hispanic | 2,701 | 11,345,274 | 23.81 (22.91-24.71) | 47.35 (45.48-49.22) |
|  | Non-Hispanic | 169,732 | 145,854,969 | 116.37 (115.82-116.92) | 83.99 (83.58-84.4) |
|  | Northeast | 65,032 | 50,644,436 | 128.41 (127.42-129.4) | 87.92 (87.23-88.6) |
|  | Midwest | 57,960 | 52,070,753 | 111.31 (110.4-112.22) | 86.09 (85.38-86.8) |
|  | South | 28,853 | 28,127,022 | 102.58 (101.4-103.76) | 84.66 (83.67-85.65) |
|  | West | 21,097 | 26,358,032 | 80.04 (78.96-81.12) | 64.21 (63.33-65.09) |
|  | Metropolitan | 138,126 | 134,565,602 | 102.65 (102.1-103.19) | 79.89 (79.46-80.32) |
|  | Non-metropolitan | 34,816 | 22,634,641 | 153.82 (152.2-155.43) | 99.72 (98.65-100.8) |
| **Q2** | All | 164,728 | 147,061,859 | 112.01 (111.47-112.55) | 88.11 (87.67-88.54) |
|  | Female | 67,723 | 74,637,890 | 90.74 (90.05-91.42) | 61.65 (61.17-62.12) |
|  | Male | 97,005 | 72,423,969 | 133.94 (133.1-134.78) | 121.48 (120.7-122.25) |
|  | American Indian/Alaska Native | 988 | 2,056,153 | 48.05 (45.05-51.05) | 65.05 (60.79-69.31) |
|  | Asian/Pacific Islander | 4,645 | 10,131,729 | 45.85 (44.53-47.16) | 49.08 (47.65-50.5) |
|  | Black/African American | 16,779 | 17,872,021 | 93.88 (92.46-95.3) | 105.97 (104.32-107.61) |
|  | White | 142,316 | 117,001,956 | 121.64 (121-122.27) | 88.54 (88.07-89) |
|  | Hispanic | 6,256 | 19,249,412 | 32.5 (31.69-33.31) | 58.55 (57.05-60.05) |
|  | Non-Hispanic | 157,779 | 127,812,447 | 123.45 (122.84-124.05) | 89.79 (89.34-90.24) |
|  | Northeast | 33,671 | 26,527,554 | 126.93 (125.57-128.28) | 92.15 (91.15-93.15) |
|  | Midwest | 64,780 | 48,489,121 | 133.6 (132.57-134.63) | 100.36 (99.58-101.15) |
|  | South | 29,464 | 26,914,531 | 109.47 (108.22-110.72) | 89.06 (88.03-90.1) |
|  | West | 36,813 | 45,130,653 | 81.57 (80.74-82.4) | 69.47 (68.75-70.19) |
|  | Metropolitan | 128,984 | 124,078,590 | 103.95 (103.39-104.52) | 84.79 (84.32-85.26) |
|  | Non-metropolitan | 35,744 | 22,983,269 | 155.52 (153.91-157.13) | 103.23 (102.14-104.33) |
| **Q3** | All | 192,540 | 170,122,659 | 113.18 (112.67-113.68) | 93.84 (93.42-94.27) |
|  | Female | 79,310 | 86,652,319 | 91.53 (90.89-92.16) | 66.57 (66.1-67.04) |
|  | Male | 113,230 | 83,470,340 | 135.65 (134.86-136.44) | 127.91 (127.15-128.67) |
|  | American Indian/Alaska Native | 1,695 | 3,511,557 | 48.27 (45.97-50.57) | 68.11 (64.72-71.5) |
|  | Asian/Pacific Islander | 6,832 | 10,977,717 | 62.24 (60.76-63.71) | 64.18 (62.64-65.73) |
|  | Black/African American | 28,644 | 29,183,620 | 98.15 (97.01-99.29) | 106.84 (105.57-108.11) |
|  | White | 155,369 | 126,449,765 | 122.87 (122.26-123.48) | 93.68 (93.21-94.15) |
|  | Hispanic | 13,628 | 33,042,141 | 41.24 (40.55-41.94) | 70.7 (69.47-71.93) |
|  | Non-Hispanic | 177,949 | 137,080,518 | 129.81 (129.21-130.42) | 96.25 (95.8-96.71) |
|  | Northeast | 22,298 | 14,280,843 | 156.14 (154.09-158.19) | 128.75 (127.04-130.46) |
|  | Midwest | 27,595 | 24,011,683 | 114.92 (113.57-116.28) | 93.36 (92.24-94.48) |
|  | South | 83,612 | 73,656,500 | 113.52 (112.75-114.29) | 91.71 (91.08-92.34) |
|  | West | 59,035 | 58,173,633 | 101.48 (100.66-102.3) | 87.72 (87.01-88.44) |
|  | Metropolitan | 156,019 | 146,235,647 | 106.69 (106.16-107.22) | 91.08 (90.62-91.53) |
|  | Non-metropolitan | 36,521 | 23,887,012 | 152.89 (151.32-154.46) | 108.78 (107.64-109.92) |
| **Q4** | All | 176,083 | 163,186,383 | 107.9 (107.4-108.41) | 92.79 (92.35-93.23) |
|  | Female | 71,177 | 82,596,622 | 86.17 (85.54-86.81) | 65.57 (65.08-66.05) |
|  | Male | 104,906 | 80,589,761 | 130.17 (129.39-130.96) | 126.2 (125.42-126.98) |
|  | American Indian/Alaska Native | 975 | 2,514,313 | 38.78 (36.34-41.21) | 53.18 (49.69-56.67) |
|  | Asian/Pacific Islander | 3,349 | 7,891,097 | 42.44 (41-43.88) | 50.26 (48.51-52.01) |
|  | Black/African American | 24,952 | 29,405,526 | 84.85 (83.8-85.91) | 96.93 (95.69-98.17) |
|  | White | 146,807 | 123,375,447 | 118.99 (118.38-119.6) | 93.82 (93.33-94.3) |
|  | Hispanic | 26,279 | 53,109,349 | 49.48 (48.88-50.08) | 74.48 (73.56-75.4) |
|  | Non-Hispanic | 149,324 | 110,077,034 | 135.65 (134.97-136.34) | 97.63 (97.13-98.13) |
|  | Northeast | 4,163 | 2,850,339 | 146.05 (141.62-150.49) | 137.12 (132.91-141.33) |
|  | Midwest | 11,782 | 12,037,705 | 97.88 (96.11-99.64) | 82.88 (81.37-84.4) |
|  | South | 133,677 | 121,636,343 | 109.9 (109.31-110.49) | 92.81 (92.31-93.31) |
|  | West | 26,461 | 26,661,996 | 99.25 (98.05-100.44) | 93.25 (92.12-94.39) |
|  | Metropolitan | 143,195 | 140,573,123 | 101.87 (101.34-102.39) | 89.6 (89.14-90.07) |
|  | Non-metropolitan | 32,888 | 22,613,260 | 145.44 (143.86-147.01) | 110.52 (109.3-111.74) |
